# Optical coherence tomography angiography analysis of changes in the foveal avascular zone in eyes with diabetic macular edema treated with intravitreal anti-vascular endothelial growth factor

**DOI:** 10.1186/s40942-022-00406-z

**Published:** 2022-08-26

**Authors:** Albert John Bromeo, Patricia Grulla-Quilendrino, Ruth Camille Antolin, Emil Joshua John Salcedo, Cheryl A. Arcinue, Ralph Anthony De Jesus, Amadeo Veloso

**Affiliations:** 1 Asian Eye Institute, Makati, Philippines; 2 Department of Ophthalmology and Visual Sciences, Philippine General Hospital, University of the Philippines, Manila, Philippines

**Keywords:** Diabetic macular ischemia, Foveal avascular zone, Optical coherence tomography angiography, Anti-VEGF, Diabetic macular edema

## Abstract

**Background:**

To analyze the changes in foveal avascular zone (FAZ) area, perimeter, and circularity in the superficial (SCP) and deep (DCP) capillary plexuses in eyes with diabetic macular edema (DME) treated with intravitreal anti-VEGF using optical coherence tomography angiography (OCTA).

**Methods:**

This prospective observational study included 56 eyes from 32 patients with DME that received intravitreal anti-VEGF. OCTA images were obtained at baseline and 1, 3, and 6 months of follow-up. The outcome measures were FAZ area, perimeter, and circularity in both the SCP and DCP, as well as central subfield thickness (CST) and best-corrected visual acuity (BCVA).

**Results:**

The mean number of intravitreal anti-VEGF injections received during the observation period was 4.60 ± 0.82 (range: 3–6). The FAZ area, perimeter, and circularity were statistically unchanged at all observation points in both the SCP (p = 0.772, p = 0.405, p = 0.157, respectively) and the DCP (p = 0.620, p = 0.769, p = 0.481, respectively). Despite having no change in the FAZ parameters, there was still a statistically significant decrease in CST (p < 0.001) as well as a statistically significant increase in BCVA (p = 0.004) during the observation period.

**Conclusions:**

The FAZ area, perimeter, and circularity in the SCP and DCP as measured by OCTA remained stable during the first 6 months of intravitreal anti-VEGF therapy in eyes with DME. While there were no significant changes in the FAZ, treatment with intravitreal anti-VEGF still resulted in decreased CST and improved BCVA.

## Background

Diabetic retinopathy is a major microvascular complication of diabetes mellitus and is one of the leading causes of preventable blindness in the world [1, 2]. Chronic hyperglycemia leads to changes in blood flow, ischemia, increased vascular endothelial growth factor (VEGF) expression, production of oxygen-free radicals, endothelial and pericyte dysfunction, and inflammation, which in turn leads to the two major causes of vision loss in diabetes—diabetic macular ischemia (DMI) and diabetic macular edema (DME) [1–3].

While significant strides have been made in the diagnosis and treatment of DME, particularly with the widespread use of optical coherence tomography (OCT), the same could not be said for DMI. DMI is clinically defined by the enlargement of the foveal avascular zone (FAZ) and paramacular area of capillary nonperfusion [4]. While DME is a well-known cause of central vision loss, studies have shown that about 41% of patients have some evidence of DMI and that visual function is affected in those with moderate to severe macular ischemia [4]. In addition, there is evidence that patients with DMI at baseline progressed earlier to neovascular forms of diabetic retinopathy during the course of intravitreal anti-VEGF therapy for DME [5]. Thus, evaluation of DMI at baseline at initiation of anti-VEGF therapy for DME has clinical importance in terms of evaluating both response of visual function and progression of retinopathy.

OCT angiography (OCTA) is a novel, noninvasive diagnostic modality that allows visualization of the retinal vasculature and microstructure. OCTA images are generated through detection of differences in the reflectivity of erythrocytes within the retinal blood vessels during successive OCT B-scans. The images are analyzed and then reconstructed to create depth-resolved and high-resolution volumetric angiograms [6]. OCTA can demonstrate and quantify the superficial and deep vascular plexuses, visualize impaired capillary perfusion and neovascularization, as well as provide information on the dimensions of the FAZ—all accomplished without the need for intravenous injection of fluorescein dye [7].

The main objective of this study is to analyze the changes in the FAZ area, perimeter, and circularity in the SCP and DCP in eyes with DME treated with intravitreal anti-VEGF therapy using OCTA. In addition, to determine the relationship of these changes with changes in central subfield thickness (CST) on OCT and best-corrected visual acuity (BCVA).

## Methods

### Study population

This was a single center, prospective, observational cohort study conducted at the Asian Eye Institute in Makati, Philippines. The Institutional Review Board of the St. Cabrini Medical Center/Asian Eye Institute gave approval for the study, and subjects were recruited from the retina service of the institution. All subjects provided written informed consent. The study was completed under the tenets of the Declaration of Helsinki.

Inclusion criteria included eyes with DME as diagnosed by a retina specialist and with standard 3 mm spectral-domain OCT planned to undergo intravitreal anti-VEGF injection. Exclusion criteria included presence of other retinal diseases such as age-related macular degeneration and retinal vascular disease, presence of media opacity precluding adequate assessment of the retina and/or producing poor image quality on OCT, and inability to complete the prescribed course of intravitreal anti-VEGF.

### Scanning protocol

OCTA was performed with the Carl Zeiss Meditec Inc. Cirrus 5000 through the AngioPlex module, Software Version 11.0.0.29946 (Carl Zeiss Meditec, Inc., Dublin, CA, USA) imaged by a trained ophthalmic technician. A standard macular cube OCT scan and OCT angiography scan were performed with the scanning area captured in 3 × 3 mm sections.

**Fig. 1:**
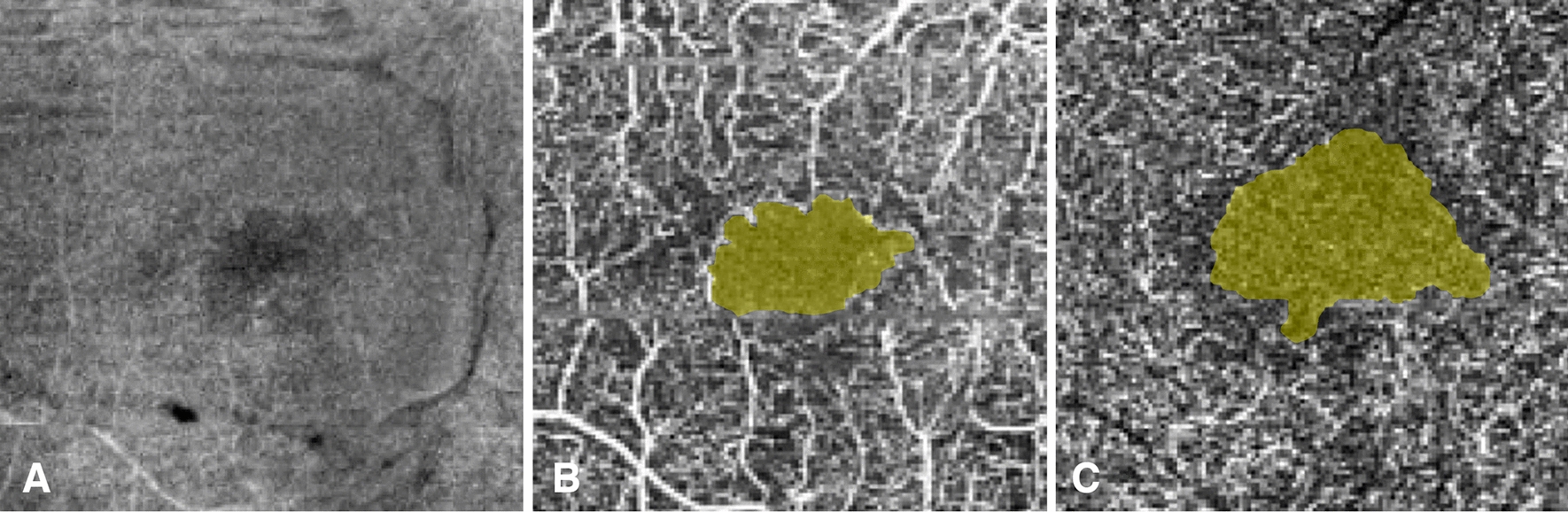
Representative images of the fovea in the SCP and DCP. **A** The fovea was defined in the 3 mm en face OCT image as the central 1 mm in the ETDRS grid. The FAZ was defined as the area encompassing the central fovea where there are no vessels seen on OCTA for both the **B** SCP and **C** DCP. The border of the FAZ was automatically calculated using the Angioplex algorithm

**Fig. 2:**
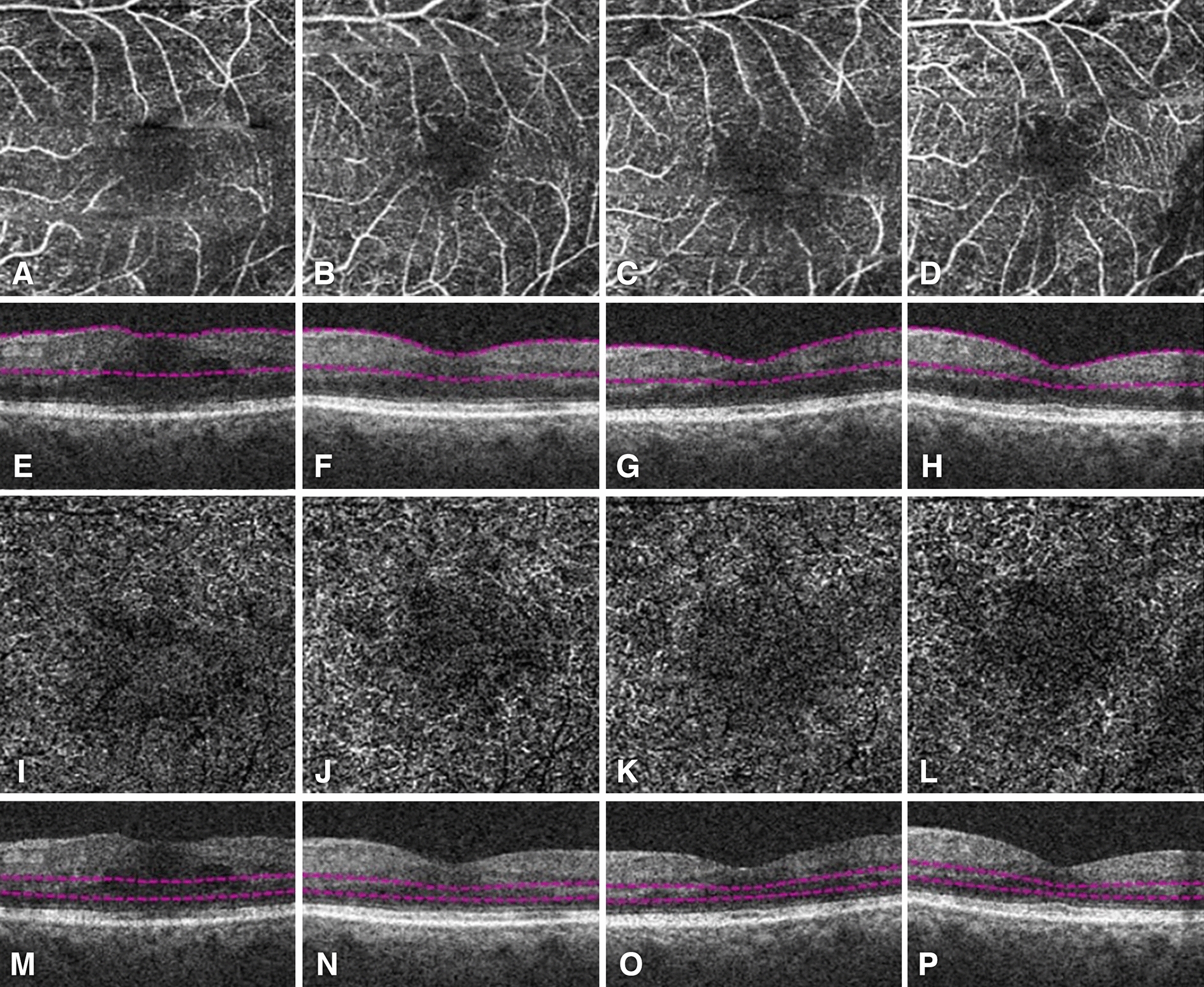
OCTA (**A**–**D**, **I**–**L**) and corresponding structural OCT (**E**–**H**, **M**–P) images of a patient with DME who underwent intravitreal anti-VEGF therapy. The FAZ in the SCP (**A**–**D**) and DCP (**I**–**L**) was imaged using OCTA at baseline (**A**, **E**, **I**, **M**), month 1 (**B**, **F**, **J**, **N**), month 3 (**C**, **G**, **K**, **O**), and month 6 (**D**, **H**, **L**, **P**)

OCTA images were segmented into the superficial capillary plexus (SCP) and deep capillary plexus (DCP) according to the software algorithm. In cases where there was segmentation error due altered retinal contour in DME, automatic segmentation lines were adjusted manually to fit the following criteria: the SCP was calculated between the internal limiting membrane and a line 15 μm deep to the outer border of the inner plexiform layer, while the DCP was calculated between the line offset 15 μm deep to the inner plexiform layer and a line offset 70 μm deep to the outer boundary of the outer plexiform layer. For each visit, the FAZ was delineated automatically by the Angioplex software. The FAZ was defined as the area encompassing the central fovea where there are no clear and demarcated vessels seen on OCTA (Fig. 1). The following parameters were then automatically calculated with the native software algorithm: FAZ area, FAZ perimeter, and FAZ circularity in both the SCP and DCP. Eyes with significant image distortion or presence of artifacts preventing measurement of the FAZ by the Angioplex software were excluded. BCVA and CST were also obtained. The patients underwent OCT and OCTA imaging at baseline, 1 month, 3 months, and 6 months of follow-up (Fig. 2).

### Statistical analysis

Statistical analysis was performed using IBM^®^ SPSS^®^ Statistics (version 28; SPSS, Inc., Chicago, IL, USA). Paired t-test and analysis of variance (ANOVA) was used for quantitative data analysis between parameters over successive observation points. A p value of less than 0.05 was considered significant.

## Results

### Baseline characteristics

A total of 56 eyes from 32 patients, including 18 males and 14 females, with a mean age of 54.07 ± 7.56 years were included in this study. The mean number of injections was 4.60 ± 0.82 (range, 3–6). The injected anti-VEGF drug was bevacizumab in 44 eyes, ranibizumab in 2 eyes, aflibercept in 2 eyes, and a mix of aflibercept and bevacizumab in 8 eyes. The clinical stage of DR in the majority of eyes was severe non-proliferative DR. The baseline characteristics are given in Table 1.

**Table 1:** Baseline characteristics of eyes of patients with DME receiving intravitreal anti-VEGF therapy

Characteristic	n (%)
Sex	
Male	18 (56%)
Female	14 (44%)
Number of anti-VEGF injections	
3	4 (7%)
4	20 (36%)
5	21 (37%)
6	11 (20%)
Anti-VEGF agent used	
Bevacizumab	44 (78%)
Ranibizumab	2 (4%)
Aflibercept	2 (4%)
Mixed	8 (14%)
Severity of diabetic retinopathy (ETDRS)	
NPDR mild	2 (4%)
NPDR moderate	16 (28%)
NPDR severe	20 (36%)
PDR early	12 (21%)
PDR high risk	6 (11%)
Underwent panretinal photocoagulation	
Yes	27 (48%)
No	29 (52%)

### Changes in FAZ area, perimeter, and circularity

Table 2 shows FAZ parameters in both the SCP and DCP, as well as CST and BCVA, at baseline and 1, 3, and 6 months following intravitreal anti-VEGF therapy. The mean FAZ area in the SCP showed a slightly increasing trend during month 1 and 3 of intravitreal anti-VEGF. At month 6, the mean area decreased from a baseline of 0.46 ± 0.29 mm^2^ to 0.44 ± 0.17 mm^2^ (p = 0.502). The mean area in the DCP showed a similar trend—slightly increasing during month 1 and 3 then decreasing at month 6. However, these changes in FAZ area in both the SCP and DCP were not statistically significant (*p* = 0.772 and *p* = 0.620, respectively).

**Table 2:** FAZ parameters, CST, and BCVA at baseline, month 1, month 3, and month 6 following intravitreal anti-VEGF therapy

Parameter	Baseline	Month 1	Month 3	Month 6	*p* value
FAZ in SCP					
Area (mm^2^)	0.46 ± 0.29	0.48 ± 0.20	0.48 ± 0.20	0.44 ± 0.17	0.772
Perimeter (mm)	3.08 ± 0.95	3.24 ± 0.81	3.40 ± 1.44	3.13 ± 0.89	0.405
Circularity	0.61 ± 0.08	0.62 ± 0.08	0.64 ± 0.08	0.64 ± 0.08	0.157
FAZ in DCP					
Area (mm^2^)	0.81 ± 0.27	0.83 ± 0.16	0.84 ± 0.18	0.79 ± 0.16	0.620
Perimeter (mm)	3.71 ± 1.30	3.85 ± 0.97	3.95 ± 1.22	3.83 ± 1.12	0.769
Circularity	0.75 ± 0.18	0.74 ± 0.18	0.73 ± 0.17	0.70 ± 0.16	0.481
CST (μm)	367.00 ± 121.81	323.65 ± 83.32	302.08 ± 49.51	292.17 ± 58.83	< 0.001
BCVA (ETDRS)	60.40 ± 19.85	63.35 ± 19.87	67.92 ± 19.48	72.94 ± 14.92	0.004

The mean FAZ perimeter in the SCP showed an increasing trend in month 1 and month 3, decreasing slightly in month 6 (but still increased compared to baseline). This trend in changes in the FAZ perimeter in the SCP is echoed in the DCP. However, the changes in FAZ perimeter in both the SCP and DCP were again not statistically significant (*p* = 0.405 and *p* = 0.769, respectively).

The mean FAZ circularity in the SCP showed an increasing trend from baseline to month 6, representing the FAZ approaching a more perfectly circular shape. This trend is not reflected in the DCP, whose results show a decreasing trend from baseline to month 6. However, once again the changes in the FAZ circularity in both the SCP and DCP were not statistically significant (*p* = 0.157 and *p* = 0.481, respectively).

**Fig. 3:**
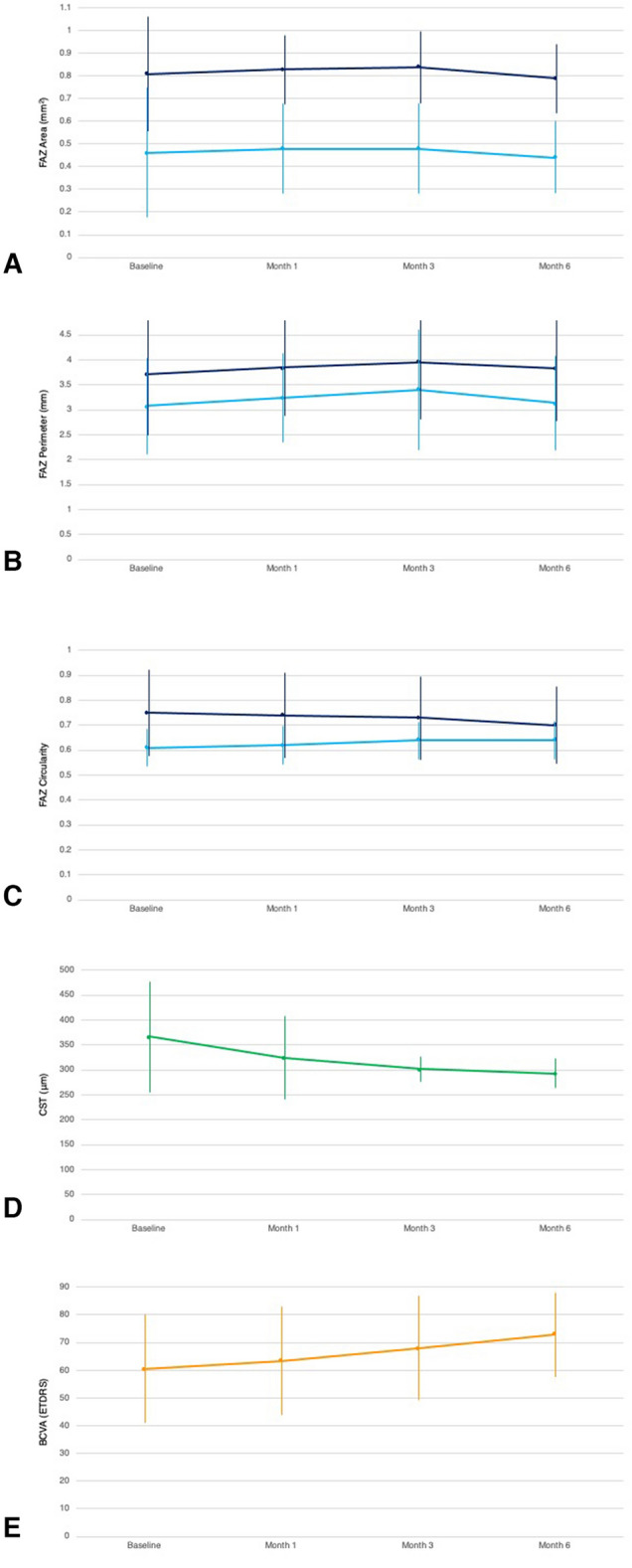
Changes in mean FAZ parameters as well as CST and BCVA following intravitreal anti-VEGF therapy. The FAZ area (**A**), perimeter (**B**), and circularity (**C**) in both the SCP (light blue) and DCP (dark blue) remain statistically unchanged at all observation points. In contrast, CST (**D**) shows a statistically significant decreasing trend. Despite the unchanged FAZ parameters, BCVA (**E**) still shows a statistically significant increasing trend over all observation points

The trends in the mean FAZ area, perimeter, and circularity during the measurement points are shown in Fig. 3.

### Association with changes in CST and BCVA

Our study also determined the association of changes in the FAZ area, perimeter, and circularity with changes in CST and BCVA. The mean CST showed a statistically significant decreasing trend in all measurement points (*p* < 0.001), decreasing from a baseline of 367.00 ± 121.81 µm to 292.17 ± 58.83 µm at month 6. The mean BCVA showed a statistically significant increasing trend in all measurement points (p = 0.004), increasing from a baseline of 60.40 ± 19.85 ETDRS letters to 72.94 ± 14.92 ETDRS letters at month 6. Thus, while there were no significant changes in the FAZ parameters measured, there were significant changes in both the mean CST and BCVA during the course of intravitreal anti-VEGF injections in these eyes.

## Discussion

Our study showed that the area, perimeter, and circularity of the FAZ in both the SCP and DCP as demonstrated by OCTA remained statistically unchanged during the first 6 months following intravitreal anti-VEGF therapy in eyes with DME. While there have been past studies documenting the changes in the FAZ area after intravitreal anti-VEGF injection, to our knowledge, this is the first study to also document the changes in FAZ perimeter and circularity as calculated by OCTA. Various techniques used to visualize the FAZ include intravenous fluorescein angiography (FA), OCTA, and more recently, retinal function imager technology [8–10]. As the use of OCTA in clinical practice becomes more widespread, it becomes important to determine the clinical value of the various parameters calculated by the various OCTA software.

The results of our study are consistent with previous studies showing that the FAZ area remains statistically unchanged after intravitreal anti-VEGF therapy [11–14]. Several factors that lead to FAZ changes include worsening severity of DR, aging, and smoking [8, 15]. Increased FAZ area has been reported in cases with DR following injection with anti-VEGF [8, 16]. Feucht et al. reported that enlargement of the FAZ may occur about 6 to 8 weeks after one intravitreal injection of 1.25 mg of bevacizumab in patients with macular edema both secondary to NPDR and BRVO. However, the effect may be transient with the effect vanishing within weeks and only noted if the retina is examined early after injection [17]. On the other hand, Gill et al. noted that FAZ area as measured using OCTA decreased over time in both observed and treated eyes with DME [18]. Thus, while there have been some studies reporting a progressive increase in the FAZ area after intravitreal anti-VEGF therapy, this finding was not established in other studies [14].

Our study also showed that the FAZ perimeter followed the trend of the FAZ area and also remained statistically unchanged after intravitreal anti-VEGF therapy. This finding is unsurprising given the mathematical relationship of the formulas for the area and perimeter of a circle, roughly the shape of the FAZ [6]. The circularity of the FAZ also did not show any statistically significant change throughout the course of intravitreal anti-VEGF, although the SCP showed an increasing trend (approaching a more perfect circle) while the DCP showed a decreasing trend (approaching a more polygonal shape than a circle). Previous studies documenting the utility of circularity ratio in diabetic retinopathy have emphasized its promise in monitoring disease progression and detecting response to treatment, showing statistically significant changes on increasing clinical severity of DR [19]. While our findings demonstrate otherwise, our small sample size or the limited observation period may account for the nonsignificant findings.

Measuring various parameters of the FAZ is used in the clinical evaluation and diagnosis of DMI. The effect of DME on the FAZ and its interaction with anti-VEGF and macular thickness makes analysis more difficult in these eyes. The anatomical swelling in eyes with DME is a factor in widening the FAZ by pushing the capillaries centrifugally [20]. The use of anti-VEGF drugs is theorized to be beneficial in patients with DMI, but so far, the literature has reported negative results [21, 22]. The effect of macular ischemia on visual outcomes remains poorly understood. Channa et al. reported that there was no significant association between severe macular ischemia and poor visual outcomes regardless of the presence or absence of residual macular edema [13]. Bates et al. reported that decreased macular edema as a result of anti-VEGF treatment allows the centripetal move of the FAZ, correlating with improved retinal structure and macular circulation [9]. On the other hand, Douvali et al. reported that macular ischemia may have a negative impact on functional outcomes after intravitreal anti-VEGF therapy in patients with DME but has no effect on anatomical outcomes [14]. Thus, the results of our study as well as the findings in previous studies highlight the current paucity in treatment options for management of DMI.

Our study also showed that while there was no significant change in the FAZ parameters measured on OCTA, treatment with intravitreal anti-VEGF was still associated with decreased CST and increased BCVA. DME has been well-known to respond to intravitreal anti-VEGF therapy with treatment having been shown to result in an increase in visual acuity [23]. Thus, while treatment with intravitreal anti-VEGF has no apparent effect on the FAZ and DMI, improved visual outcomes are still achieved through treatment of DME.

Our study has several limitations. The study has a small number of eyes with different stages of DR. The study had a limited observation period of 6 months which comprises a very short follow-up and may have been inadequate to demonstrate changes in the FAZ. The observational study did not determine the effect of other variables such as number of anti-VEGF injections, level of control of diabetes, clinical severity of DR, and effect of panretinal photocoagulation on the FAZ parameters. The small sample size did not allow for a statistically significant comparison between the effect of the different anti-VEGF drugs (bevacizumab, ranibizumab, and aflibercept). Lastly, the effect of other OCT biomarkers such as disorganization of the inner retinal layers (DRIL), cystoid changes, and hyperreflective elements in the retinal layers were not evaluated.

## Conclusions

Our study showed that the FAZ area, perimeter, and circularity in the SCP and DCP as measured by OCTA remained stable during the first 6 months of intravitreal anti-VEGF therapy in eyes with DME. While there were no significant changes in the FAZ, treatment with intravitreal anti-VEGF still resulted in decreased CST and improved BCVA. Future studies with a larger sample size and longer follow-up period may be needed to confirm our findings. Furthermore, a randomized controlled trial may be necessary to control the effect of other confounding variables.

## Data Availability

The datasets used and/or analysed in this current study are available from the corresponding author on reasonable request.

## Abbreviations

BCVA: Best corrected visual acuity
CST: Central subfield thickness
DCP: Deep capillary plexus
DME: Diabetic macular edema
DMI: Diabetic macular ischemia
ETDRS: Early Treatment Diabetic Retinopathy Study
FAZ: Foveal avascular zone
OCT: Optical coherence tomography
OCTA: Optical coherence tomography angiography
SCP: Superficial capillary plexus
VEGF: Vascular endothelial growth factor
